# Resource Partitioning between Two Piranhas (*Serrasalmus gibbus* and* Serrasalmus rhombeus*) in an Amazonian Reservoir

**DOI:** 10.1155/2017/8064126

**Published:** 2017-11-20

**Authors:** Júlio C. Sá-Oliveira, Stephen F. Ferrari, Huann C. Gentil Vasconcelos, Andrea S. Araujo, Carlos E. Costa Campos, Claudio A. Gellis Mattos-Dias, Amanda A. Fecury, Euzébio Oliveira, Raimundo N. G. Mendes-Junior, Victoria J. Isaac

**Affiliations:** ^1^Ichthyology and Limnology Laboratory, Federal University of Amapá (UNIFAP), Campus Universitário Marco Zero do Equador, Rod. Juscelino Kubitscheck, KM-02, 68903-419 Macapá, AP, Brazil; ^2^Department of Ecology, Federal University of Sergipe, Avenida Marechal Rondon s/n Rosa Elze, 49000-100 São Cristóvão, SE, Brazil; ^3^Zoology Laboratory, Federal University of Amapá (UNIFAP), Campus Universitário Marco Zero do Equador, Rod. Juscelino Kubitscheck, KM-02, 68903-419 Macapá, AP, Brazil; ^4^Herpetology Laboratory, Universidade Federal do Amapá (UNIFAP), Rod. Juscelino Kubitscheck, KM-02, 68903-419 Macapá, AP, Brazil; ^5^Instituto Federal do Amapá, Rod. BR-210, Km 03, s/n, Brasil Novo. 68909-398 Macapá, AP, Brazil; ^6^Departamento de Ciências Biológicas e da Saúde, Federal University of Amapá (UNIFAP), Campus Universitário Marco Zero do Equador, Rod. Juscelino Kubitscheck, KM-02, 68903-419 Macapá, AP, Brazil; ^7^Federal University of Pará (UFPA), Campus Guamá, 01 Rua Augusto Corrêa, Guamá, 66075-110 Belém, PA, Brazil; ^8^Cajari River Extractive Reserve, Instituto Chico Mendes de Conservação da Biodiversidade (ICMBio), Rua Leopoldo Machado, 1126, Centro, Macapá, AP, Brazil; ^9^Fisheries Biology Laboratory, Federal University of Pará (UFPA), Campus Guamá, 01 Rua Augusto Corrêa, Guamá, 66075-110 Belém, PA, Brazil

## Abstract

The exploitation of resources by closely related species with similar niches may be mediated by differences in activity patterns, which may vary in nycthemeral scale and seasonal scale. Piranhas* Serrasalmus gibbus* and* Serrasalmus rhombeus* are Neotropical predators that occur sympatrically in many environments of the Amazon basin. To evaluate the strategies adopted by these two species in a restricted environment (a reservoir), nycthemeral and seasonal samples were made, identifying the composition of the diet and their activity patterns. A total of 402 specimens were collected: 341* S. gibbus* and 61* S. rhombeus*. Both species fed themselves primarily on fish, with some seasonal variation being found in* S. gibbus* during the flood season, when plant material was consumed. There was considerable temporal overlap in the foraging behavior of the two species, although* S. rhombeus* presented a bimodal pattern of abundance over the 24-hour cycle.* S. rhombeus* was more active during the nighttime, between dusk and early morning, whereas* S. gibbus* was active throughout the nycthemeral cycle. These findings indicate low levels of competition between the two species, which allowed for a considerable overlap in nighttime foraging, following distinct nycthemeral patterns of foraging activity and allowing their coexistence.

## 1. Introduction

In impacted environments, such as reservoirs, limitations of resource availability may be one of the principal factors determining the composition of fish communities [1] and resource partitioning [2]. Studies of resource partitioning can help elucidate how populations of closely related species can coexist under such conditions, even when one species dominates the system and can, at least theoretically, exclude the others [3, 4]. Despite this, observations in the wild, especially in the tropics, indicate that these species can coexist, even when exploiting the same resources [4].

Studies of the feeding ecology of fish are essential for the understanding of trophic relationships among species in aquatic systems [5]. In general, trophic studies of fish have shown that the same resource may be exploited by a number of different species and that each species may exploit a number of different feeding resources within a given area [6–9]. Resource partitioning tends to involve three principal axes of the niche, time, space, and foraging behavior, which may determine the potential for the coexistence of populations within a given area [3]. In the spatial dimension, for example, the use of distinct microhabitats may permit the development of extremely narrow niches which minimize interspecific competition [10]. When feeding resources are abundant, niches may overlap without affecting the competing species [3]. The stable coexistence of species limited by the same resource is possible if the interaction of conspecifics predominates over that between species [11], which may account for the coexistence of species limited by the same resource without effective niche partitioning [12].

Environmental factors also influence resource partitioning by affecting the availability of resources directly, as in the case of the hydrological cycle in aquatic systems [2, 13]. The niche of a species may vary over time, according to modifications of the environment and the availability of resources [14], and resource dynamics may lead to the adoption of more specialist or generalist niches within a given area [15], with species adjusting to the presence of competitors [16].

Piranha species are phenotypically homogeneous and mostly exploit similar resources, resulting in intense competition [5], although this competition may be mediated by the relative abundance of resources found in tropical environments [17]. Piranhas of the genus* Serrasalmus* have been studied in a number of Brazilian rivers [18–24], although no data are available for the Araguarí-Amapá basin.

The gibbus piranha,* Serrasalmus gibbus* Castelnau 1855, is a carnivorous species of the family Serrasalmidae, which feeds on fish, insects, and other invertebrates, and is found primarily along the margins of rivers and lakes [25]. The black piranha,* Serrasalmus rhombeus* Linnaeus 1766 (Serrasalmidae), is also carnivorous. It is the largest piranha species, with adults reaching 50 cm in length, and is considered to be one of the most successful fish species in Amazonian reservoirs [25].

In addition to being closely related,* S. gibbus* and* S. rhombeus* are sympatric, although little is known about the ecological variables that support their coexistence. Given this, the present study analyzed the nycthemeral and seasonal variation in the diets of* S. gibbus* and* S. rhombeus* in the Coaracy Nunes reservoir in Amapá, northern Brazil, in order to understand the resource partitioning in these closely related carnivores.

## 2. Materials and Methods

### 2.1. Study Area

**Figure 1:**
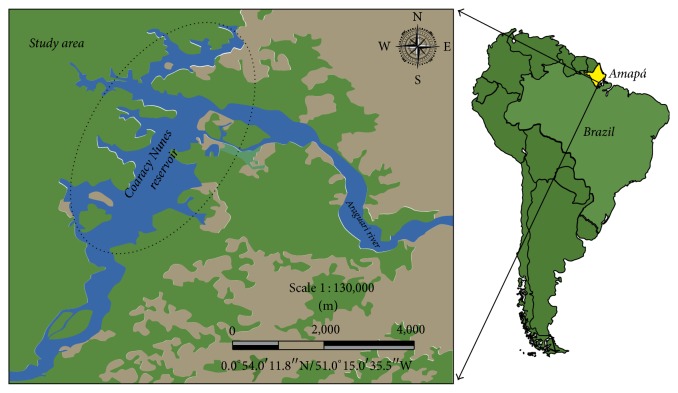
Study area: reservoir of the Coaracy Nunes hydroelectric power station in Ferreira Gomes, Amapá, Brazil.

The study area is located in the middle Araguarí River basin (Amapá-Brazil) in the Coaracy Nunes reservoir (0°57′1.09′′N/51°14′50.79′′W, 0°51′10.45′′N/51°17′44.38′′W, 0°52′53.80′′N/51°18′34.72′′W, and 0°52′2.89′′N/51°15′35.51′′W). The Coaracy Nunes reservoir lies between the municipalities of Ferreira Gomes and Porto Grande in the state of Amapá and is located approximately 200 km from the Atlantic Ocean. The reservoir drains a total area of 23.5 km^2^ and has a mean discharge of 976 m^3^·s^−1^, mean depth of 15 m, and a total volume of 138 Hm^3^. The local climate is typical of the Amazon basin, with a rainy season between January and June and a dry season from July to December [26–28] (Figure 1).

### 2.2. Sampling

Samples of* S. gibbus* and* S. rhombeus* were collected every two months between September 2011 and September 2012. The fish were captured in gillnets arranged in sets of increasing mesh size (2 cm, 2.5 cm, 3 cm, 4 cm, 5 cm, 8 cm, and 10 cm), varying in length from 10 m to 20 cm, and varying in height from 1.5 m to 4 m. The nets were set at 15:00 h and left until 15:00 h of the following day and were checked every three hours, that is, at 18:00 h, 21:00 h, 24:00 h, 03:00 h, 06:00 h, 09:00 h, 12:00 h, and 15:00 h, with the same standard procedure being followed on each day of fieldwork.

The captured specimens were identified, measured, and weighed, and their stomachs were removed for analysis. Each stomach was weighed, and its contents were analyzed immediately. The total length (TL, in mm) was obtained for each specimen using a caliper and measuring tape, and the total weight (TW), gutted weight (GW), and stomach weight (SW) were determined using a scale with a precision of 0.01 g, based on [29]. The sex of the specimens was determined through the inspection of the gonads.

### 2.3. Analysis of Stomach Contents

The repletion of the stomachs was determined by visual inspection and classified as (i) void (no content), (ii) partly full (25–75% full), and (iii) full (>75%), following [30]. The contents of the stomachs classified as full or partly full were identified and quantified using a stereomicroscope [31]. For the standardization of the samples, the items were grouped in broad categories, that is, fish, insect, crustacean, microcrustacean, plant, and others [31].

### 2.4. Data Analysis

The activity pattern of each species was estimated based on the relative frequency of the specimens captured at each net check (3-hour intervals). The temporal variation in activity was analyzed using a *χ*^2^ test, considering differences among the 3-hour intervals and between the nocturnal (18:00–06:00 h) and diurnal (06:00–18:00 h) periods. The lengths and weights of the males and females of each species were allocated to classes, which were tested for normality (Kolmogorov-Smirnov) and homoscedasticity (Bartlett) before being tested for differences using Student's *t*-test, with *α* = 0.05.

The analysis of stomach contents was based on the frequency of occurrence and volumetric methods [32]. The frequency of occurrence was determined by FO_*i*_ = (*n*_*i*_ × 100)/*N*, where FO_*i*_ is the frequency of occurrence of item *i* in the sample, *n*_*i*_ is the number of stomachs in the sample which contain item *I*, and *N* is the total number of stomachs containing ingesta in the samples. The volumetric method was based on the relative volume of each item in the stomach contents. The volume of the items was estimated based on the method of [33, 34]. The relative importance of each item in the diet of the species and the FO and volumetric values were combined in the index of feeding importance (IF_*i*_) of [30], calculated for the two seasons (dry and flood). The nycthemeral and seasonal variation in the frequency of occurrence and IF_*i*_ of each species were evaluated using the *G* test, with *α* = 0.05. The variation in the repletion of the stomachs and the overlap in the nycthemeral and seasonal niches of the two species were evaluated using *χ*^2^, with *α* = 0.05.

Levin's standardized index (*Bi*) was used to define trophic niche breadth [35], which was classified as low (*Bi* < 0.4), medium (0.4–0.6), or high (*Bi* > 0.6) [36]. Niche overlap was analyzed using 3 indices, which varies from 0 (no overlap) to 1 (complete overlap), representing the degree of resource partitioning. As for niche breadth, this index was classified as low (<0.4), moderate (0.4–0.6), or high (>0.6) [36]. The index was calculated in EcoSim 7.0 [37].

To evaluate whether the observed overlap was different from that expected by chance (in the absence of competition), the abundance of the different food items in the diet of each species was randomized using null models with 5000 randomizations, run in the RA3 randomization algorithm in EcoSim [38]. In this analysis, interspecific competition may be occurring when the mean trophic niche overlap is lower than that expected by chance [39].

## 3. Results

A total of 341 specimens of* S. gibbus* were collected during the present study, including 177 males (51.9% of the total) and 164 (48.1%) females, with no significant bias in the sex ratio (*χ*^2^ = 0.49, df = 1, and *p* ≥ 0.05). In the case of* S. rhombeus*, 39 (64.0%) of the 61 specimens were female and 22 (36.0%) were male, with a significant female bias (*χ*^2^ = 4.73, df = 1, and *p* = 0.02; Yates = 4.19 and *p* < 0.05).

**Figure 2:**
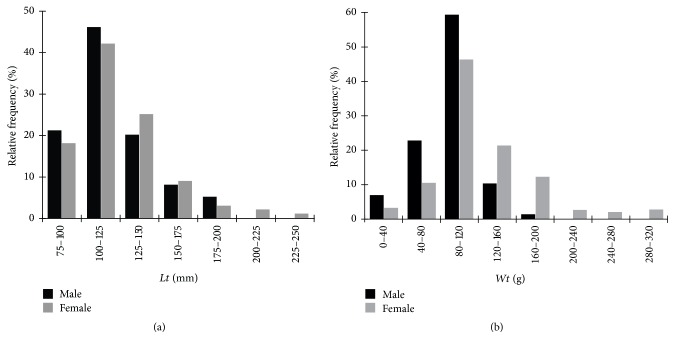
Population structure in total length (a) and weight (b) of* S. gibbus* in the Coaracy Nunes reservoir in Ferreira Gomes, Amapá (Brazil).

**Figure 3:**
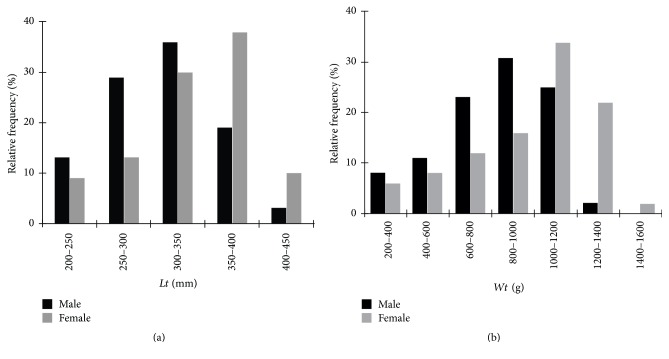
Population structure in total length (a) and weight (b) of* S. rhombeus* in the Coaracy Nunes reservoir in Ferreira Gomes, Amapá (Brazil).

The mean total length of the male* S. gibbus* specimens was 120.9 ± 25.5 mm and that of* S. rhombeus* males was 323.0 ± 59.0 mm. The female* S. gibbus* were slightly larger than the males, with a mean length of 125.0 ± 31.4 mm, whereas the female* S. rhombeus* were much smaller than the males, with a mean length of 303.6 ± 61.2 mm. The mean weight of the male* S. gibbus* (90.56 ± 29.61 g) was also lower than that of the females (131.21 ± 51.95 g), whereas that of the male* S. rhombeus* (742.0 ± 423.0 g) was slightly higher than that of the females (729.9 ± 449.7 g). The body length (*t* = 17.49, df = 105, and *p* < 0.0001) and weight (*t* = 19.58, df = 105, and *p* < 0.0001) of the two species were significantly different, although no statistical differences were found between the sexes of either species (*S. gibbus*:* t* = −0.1433, df = 116.0, and *p* > 0.05;* S. rhombeus*:* t* = −0.1294, df = 71.0, and *p* > 0.05). However, while the distributions of body lengths and weight classes were relatively similar in the two sexes in* S. gibbus* (Figure 2), the most frequent classes in the female* S. rhombeus* were higher than those in the males (Figure 3).

**Figure 4:**
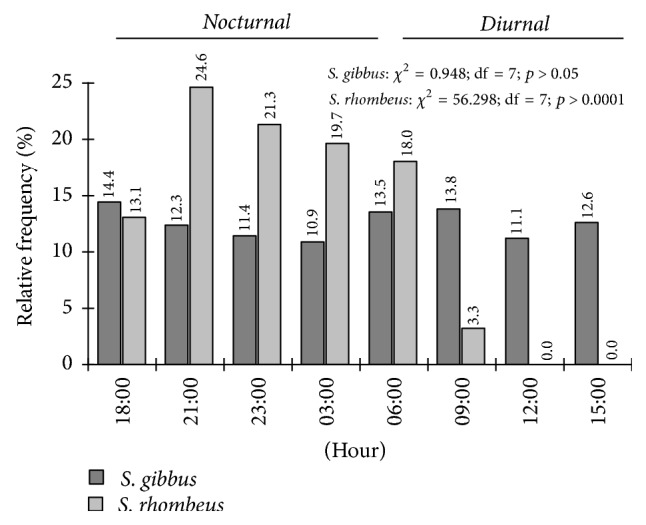
Relative frequency of the* S. gibbus* and* S. rhombeus* specimens captured per 3-hour interval in the Coaracy Nunes reservoir in Ferreira Gomes, Amapá (Brazil).

No clear nycthemeral pattern was observed in* S. gibbus* (Figure 4) either among 3-hour intervals (*χ*^2^ = 0.948; df = 7; *p* ≥ 0.05) or between nocturnal and diurnal periods (*χ*^2^ = 0.823; df = 1; *p* ≥ 0.05). By contrast,* S. rhombeus* presented a clear preference for the nocturnal period (Figure 4), peaking in the first half of the night, with highly significant differences among intervals (*χ*^2^ = 56.298; df = 7; *p* < 0.05) and between nocturnal and diurnal periods (*χ*^2^ = 22.601; df = 1; *p* < 0.05; Yates = 21.661 and *p* < 0.05), but there was no difference between seasons (*χ*^2^ = 0.981; df = 1; *p* ≥ 0.05).

### 3.1. Stomach Contents and Repletion

**Figure 5:**
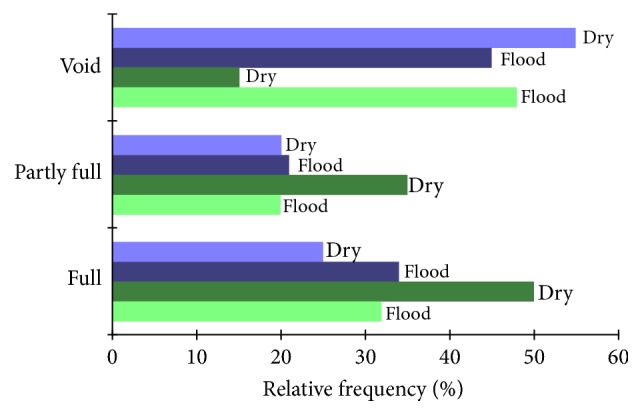
Seasonal variation in the repletion of the stomachs of the* S. gibbus* (blue) and* S. rhombeus* (green) specimens collected in the Coaracy Nunes reservoir in Ferreira Gomes, Amapá (Brazil).

A total of 137 specimens had at least partly full stomachs, of which 115 were* S. gibbus* and 22 were* S. rhombeus*. In* S. gibbus*, the void class was the most frequent in both seasons (Figure 5), followed by the full class, although there was no significant seasonal difference in either class (void: *χ*^2^ = 1.25; df = 1; *p* ≥ 0.05; full: *χ*^2^ = 1.32; df = 1; *p* ≥ 0.05). In* S. rhombeus*, the void class was the most frequent in the flood season, followed by the full class. In the dry season, the full and partly full classes were the most frequent, and while there was no seasonal variation in the full class (*χ*^2^ = 3.48; df = 1; *p* ≥ 0.05), the partly full class varied seasonally (*χ*^2^ = 5.91; df = 1; *p* < 0.05; Yates = 5.378 and *p* < 0.05).

**Figure 6:**
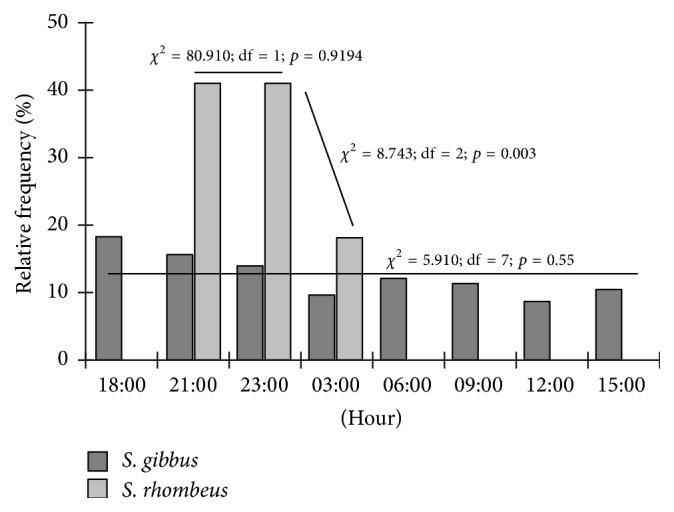
Nycthemeral variation in the relative frequency of repletion of the stomachs (at least partly full) of the* S. gibbus* and* S. rhombeus* specimens collected in the Coaracy Nunes reservoir in Ferreira Gomes, Amapá (Brazil).

No nycthemeral variation was found in the frequency of* S. gibbus* specimens with at least partly full stomachs (Figure 6). In* S. rhombeus*, however, a highly significant peak was recorded between 21:00 h and 03:00 h, with only void stomachs being recorded during the other intervals (*χ*^2^ = 194.22; df = 7; *p* < 0.05).

The two piranha species consumed five dietary items (fish, crustaceans, microcrustaceans, insects, and plants), with fish being the principal item consumed by both species, during both seasons (Table 1). The diet of* S. gibbus* was composed of fish (remains including fins, bones, scales, and muscle), crustaceans (shrimp,* Macrobrachium* sp.), insects (Odonata, Orthoptera), microcrustaceans (Isopoda), and plant matter (fragments of leaves, twigs, and seeds). The diet of* S. rhombeus* consisted of fish, crustaceans, microcrustaceans, and insects. Fish was more predominant in the diet during the dry season, with significant seasonal variation in the FO in both* S. gibbus* and* S. rhombeus* (Table 1). However, IF_*i*_ did not vary significantly between seasons in either species.

**Figure 7:**
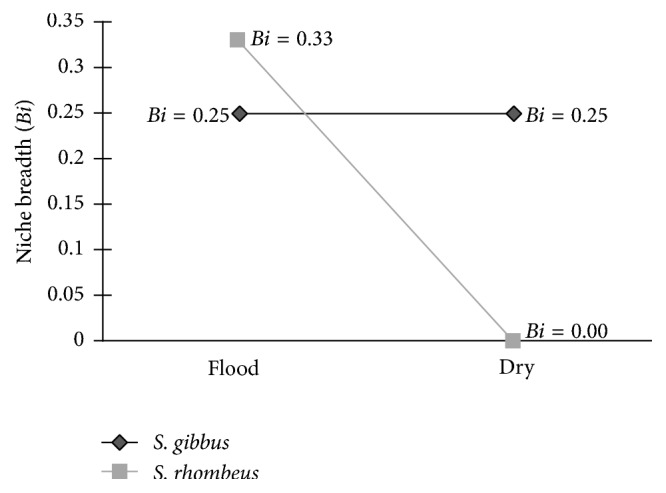
Seasonal variation in niche breadth in* S. gibbus* and* S. rhombeus* in the Coaracy Nunes reservoir in Ferreira Gomes, Amapá (Brazil).

Niche breadth did not vary seasonally in* S. gibbus* (Figure 7). In* S. rhombeus*, by contrast, while niche breadth was higher than that of* S. gibbus* in the flood season, it fell to zero in the dry season, when only fish was consumed.

A high degree of niche overlap was recorded in both the dry (*O*_*jk*_ = 0.999) and flood (*O*_*jk*_ = 0.899) seasons (Table 2). The mean observed index (0.997) was significantly higher than the simulated mean (0.241), reflecting the niche overlap of the species in this environment. The observed *C* scores were significantly higher than the expected scores, indicating that they were not random, and thus reflect biological processes. In the analysis of the nycthemeral variation, however, the observed *C* scores were significantly lower than those expected by chance in both seasons.

## 4. Discussion

One fundamental ecological difference between the two study species was the difference in their abundance, with* S. rhombeus* being far less common than* S. gibbus*. This may reflect the effects of interspecific competition on the demographic parameters of the two populations [40].

The sex ratio of* S. gibbus* was unbiased (1 : 1), although it was female-biased (64%; *n* = 39) in* S. rhombeus*. Females tend to predominate in populations when feeding resources are abundant [29, 41, 42]. When the sex ratio is balanced (1 : 1), the males compete for access to females and this reduces quantities of them [41–45]. Anderson [44] concluded that the lack of a male reduces competition for females.

The body size structure of a fish population varies as a function of recruitment and mortality, which are influenced by the biotic and abiotic variables that determine their nutritional condition [46] and birth and survival rates [47]. In the present study, however, no significant sexual dimorphism was found in either* S. gibbus* or* S. rhombeus*. While sexual dimorphism may contribute to the avoidance of intraspecific competition [48, 49], no evidence was found of this phenomenon in the study populations. However, the differences in body size between the two species may represent an important feature of interspecific niche partitioning, which may be related primarily to the differences in activity patterns, while morphologically similar species tend to occupy similar niches, implying competition for resources and eventual competitive exclusion [50, 51]. In the present case,* S. gibbus* and* S. rhombeus* are morphologically so similar that [52] originally classified them as a single species, although [53] concluded that* S. gibbus* is a valid species, based on its relatively more elongated body and larger interorbital distance in comparison with* S. rhombeus*. In fish, the trophic level is normally related to the size of the predator [54], which indicates that* S. gibbus* feeds on smaller prey than* S. rhombeus*, reducing the potential for direct competition and permitting the coexistence of the two species.

The large number of empty stomachs recorded in both species throughout the study period is characteristic of carnivorous species [55]. This situation was evident in* S. gibbus* in both seasons but different in* S. rhombeus*, in which more individuals had full stomachs, especially during the dry season, when prey are more concentrated in a small space. It is important to note that easily digested items of high nutritional value spend less time in the digestive tract, resulting in higher rates of empty stomachs [56]. One other important factor may have been the use of gillnets to capture the specimens, which may regurgitate their stomach contents in their attempt to free themselves from the net [57].

In addition to the difference in body size, niche partitioning between the two species was supported by the difference in activity patterns. While* S. gibbus* was active throughout the nycthemeral cycle,* S. rhombeus* was mainly active during the night. This difference may contribute decisively to niche partitioning [58, 59] and may have evolved in the context of the coexistence of the two species [39].

The third aspect of the coexistence of the two species was the composition of their diets. While both piranhas fed primarily on fish,* S. gibbus* fed on a greater diversity of items, including plants. The other items were consumed in varying proportions by the two piranhas, except for insects during the flood season. While insects and fish were potentially the most disputed items during the flood season, competition for these resources was probably assuaged by their abundance during this period. During the dry season,* S. rhombeus* fed exclusively on fish, and while the diet of* S. gibbus* included some other items, it also fed predominantly on fish, although the concentration of stocks during this low water period facilitated predation, which, once again, would have reduced direct competition for this resource.

The compositions of the diets of the study species were typical of piranhas [21, 60, 61]. In the present study, insects were a prominent component of the diet of both species, except for* S. rhombeus* during the dry season, confirming the relative importance of this item, as reported by other authors [19, 20, 62–64]. The predation of insects is an important aspect of the dietary plasticity of the piranhas, allowing them to adjust their intake of nutrients to the local availability of resources [31, 62, 65].

The high level of overlap (>6) in the composition of the diets of* S. gibbus* and* S. rhombeus* recorded in the present study may have been influenced by the frequent predation of fish trapped in the fishing nets, resulting in the high indices recorded for this item. It is likely that the two piranha species had preferences for different types of fish prey, although it was not possible to confirm this based on the material collected from the stomach contents, which was mostly indistinguishable. While the two piranhas inhabit the same environment and often consume the same types of food, the minor differences in their diets probably minimize competition between them [66]. In the present case, the relative abundance of resources may allow for considerable overlap in the diets of the different species, especially where temporal or spatial segregation mediates competition between them [67].

In fact, the analysis of niche overlap over the nycthemeral cycle (Pianka's index) indicated significantly lower than expected overlap, which implies an absence of competition. In the seasonal analysis, however, there was evidence that the observed patterns were not random, indicating the role of biological processes, such as competition, which may nevertheless be mediated by the relative abundance of the most consumed resources. It is important to remember that, in more complex environments, where a greater diversity of resources is available, each item may be exploited less intensively, reducing competition and favoring the coexistence of the species [68].

Overall, the results of the present study indicate that the coexistence of* S. gibbus* and* S. rhombeus* in the Coaracy Nunes reservoir is mediated by the relative abundance of feeding resources (*Leporinus af. parae*,* Leporinus affinis*,* Leporinus maculatus*,* Tometes trilobatus*,* Hemiodus unimaculatus*,* Geophagus proximus*,* Plagioscion squamosissimus*,* Satanoperca acuticeps*,* Triportheus trifurcatus*, etc.) and differences in the timing of foraging behavior, which minimize the direct competition between the two species. The two species studied represent those of greater abundance in this reservoir. The adaptive success of these predators in dammed environments promotes the reduction of fish diversity, especially those species occupying other trophic niches, which increases the initial environmental impact generated by the dam. Understanding the functional dynamics of fish trophic guilds in dammed environments can be an important step in the conservation orientation of these environments.

## Figures and Tables

**Table 1:** Frequency of occurrence (FO), volumetric frequency (VF), and index of feeding importance (IF_*i*_) of the items encountered in the stomach contents of the *S. gibbus* and *S. rhombeus* specimens captured in the dry and flood seasons in the Coaracy Nunes reservoir in Ferreira Gomes, Amapá (Brazil).

Species	Food item	Flood			Dry			*G* test	
		FO (%)	VF (%)	IF_*i*_	FO (%)	VF (%)	IA*i*	FO	IF_*i*_
*S. gibbus*	Fish	65.517	22.014	0.9671	96.429	37.118	0.9588	16.94^*∗*^	
	Crustacean	10.345	2.510	0.0174	10.714	3.857	0.0111		
	Insect	20.689	1.089	0.0151	14.286	0.843	0.0032		0.044^*∗∗*^
	Plant	3.448	0.138	0.0003	25.000	4.007	0.0268		
	Microcrustacean	3.448	0.055	0.0001	3.571	0.075	0.0001		

*S. rhombeus*	Fish	80.000	0.906	0.9747	100.000	1.000	1.000	78.70^*∗*^	0.04^*∗∗*^
	Crustacean	20.000	0.089	0.0239	0.000	0.000	0.000		
	Insect	20.000	0.004	0.0011	0.000	0.000	0.000		
	Microcrustacean	20.000	0.001	0.0003	0.000	0.000	0.000		

^*∗*^ *p* < 0.05; ^*∗∗*^*p* ≥ 0.05.

**Table 2:** Mean observed and simulated Pianka indices and their respective *p* values for the dietary overlap of *S. gibbus* and *S. rhombeus* in the Coaracy Nunes reservoir in Ferreira Gomes, Amapá (Brazil).

Parameter	Seasonal	Nycthemeral	
		Flood	Dry
(*O*_*jk*_): observed mean	0.997	0.531	0.519
(*O*_*jk*_): simulated mean	0.241	0.606	0.606
Variance of the simulated indices	0.083	0.001	0.002
*C score*: *p* (observed ≤ expected)	1.000	0.018^*∗*^	0.016^*∗*^
*C score*: *p* (observed ≥ expected)	0.000^*∗*^	0.982	0.984

^*∗*^ *p* < 0.05.

## References

[1] Benedito-Cecílio E., Agostinho A. A., Agostinho A. A., Gomes L. C. (1997). Estrutura das populações de peixes do reservatório de Segredo. *Reservatório de Segredo: bases ecológicas para o manejo*.

[2] Brandão-Gonçalves L., Lima-Junior S. E., Suarez Y. R. (2009). Feeding habits of Bryconamericus stramineus Eigenmann, 1908 (Characidae), in different streams of Guiraí River Sub-Basin, Mato Grosso do Sul, Brazil. *Biota Neotropica*.

[3] Pianka E. R. (1973). The structure of lizard communities. *Annual Review of Ecology, Evolution, and Systematics*.

[4] Giacomini H. C. (2007). Os mecanismos de coexistência de espécies como vistos pela teoria ecológica. *Oecologia Brasiliensis *.

[5] Wootton R. J. (1990). *Ecology of Teleost Fishes*.

[6] Hahn N. S., Fugi R., Andrian I. F., Thomaz S. M., Agostinho A. A., Hahn N. S. (2004). Trophic ecology of the fish assemblages. *The upper Paraná river and its floodplain physical aspects, ecology and conservation*.

[7] Mérona B. d., Rankin-de-Mérona J. (2004). Food resource partitioning in a fish community of the central Amazon floodplain. *Neotropical Ichthyology*.

[8] Pouilly M., Yunoki T., Rosales C., Torres L. (2004). Trophic structure of fish assemblages from Mamoré River floodplain lakes (Bolivia). *Ecology of Freshwater Fish*.

[9] Souto A. C. (2011). *Partilha de recursos alimentares nas assembléias de peixes do reservatório de Salto Grande (Médio rio Paranapanema SP/PR, Brasil)*.

[10] Toft C. A. (1985). Resource paratitioning in amphibians and repitiles. *Copeia*.

[11] Hartley S., Shorrocks B. (2002). A general framework for the aggregation model of coexistence. *Journal of Animal Ecology*.

[12] Begon M., Harper J. L., Townsend C. R. (1996). *Ecology. Individuals, Populations and Communities*.

[13] Berg J. (1979). Discussion of methods of investigating the food of fishes, with reference to a preliminary study of the prey of Gobiusculus flavescens (Gobiidae). *Marine Biology*.

[14] Krebs C. J. (1989). *Ecological Methodology*.

[15] Abelha M. C. F., Agostinho A. A., Goulart E. (2001). Plasticidade trófica em peixes de água doce. *Acta Scientiarum Biological Sciences*.

[16] Andersen A. N. (1992). Regulation of 'momentary' diversity by dominant species in exceptionally rich ant communities of the Australian seasonal tropics. *The American Naturalist*.

[17] Lowe-McConnell R. H. (1999). *Estudos Ecológicos de Comunidade de Peixes Tropicais*.

[18] Agostinho C. S., Marques E. E. (2001). Selection of netted prey by piranhas, *Serrasalmus spilopleura* and *Serrasalmus marginatus* (Pisces, Serrasalmidae). *Acta Scientiarum Biological Sciences*.

[19] Oliveira A. K., Alvim M. M. C., Peret A. C., Alves C. B. M. (2004). Diet shifts related to body size of the pirambeba (*Serrasalmus brandtii*) Lütken, 1875 (Osteichthyes, Serrasalminae) in the Cajuru reservoir, São Francisco river basin, Brazil. *Brazilian Journal of Biology*.

[20] Costa A. C., Salvador Junior L. F., Domingos F. F., Fonseca M. L. (2005). Alimentação da pirambeba Serrasalmus spilopleura Kner, 1858 (Characidae; Serrasalminae) em um reservatório do Sudeste brasileiro. *Acta Scientiarum. Biological Sciences*.

[21] Piorski N. M., Alves J. D. R. L., Machado M. R. B., Correia M. M. F. (2005). Alimentação e ecomorfologia de duas espécies de piranhas (Characiformes: Characidae) do lago de Viana, estado do Maranhão, Brasil. *Acta Amazônica*.

[22] Peretti D. (2006). *Alimentação e análise morfológica de quatro espécies de peixes (Astyanax altiparanae, Parauchenipterus galeatus, Serrasalmus marginatus e Hoplias aff. malabaricus) na planície de inundação do alto rio Paraná, Brasil*.

[23] Hubert N., Duponchelle F., Nuñez J., Garcia-Davila C., Paugy D., Renno J.-F. (2007). Phylogeography of the piranha genera *Serrasalmus* and *Pygocentrus*: implications for the diversification of the Neotropical ichthyofaunal. *Molecular Ecology*.

[24] De Jesus Trindade M. E., Jucá-Chagas R. (2008). Diet of two serrasalmin species, *Pygocentrus piraya* and *Serrasalmus brandtii* (Teleostei: Characidae), along a stretch of the rio de Contas, Bahia, Brazil. *Neotropical Ichthyology*.

[25] Santos G. M., Ferreira E. J. G., Zuanon J. A. S. (2006). *Peixes comerciais de Manaus*.

[26] Bezerra P. E. L., Oliveira V., Regis W. D. E., Brazão J. E. M., Gavinho I., Coutinho R. C. P. (1990). *Projeto de Zoneamento de Recursos Naturais da Amazônia Legal*.

[27] IBGE Instituto Brasileiro de Geografia e Estatística. http://www.ibge.gov.br.

[28] Sá-Oliveira J. C., Isaac-Nahum V. J. (2013). Diet Breadth and Niche Overlap Between *Hypostomus plecostomus* (Linnaeus, 1758) and *Hypostomus emarginatus* (Valenciennes, 1840) (Siluriformes) in the Coaracy Nunes Hydroelectric Reservoir, Ferreira Gomes, Amapá-Brazil. *Biota Amazônia*.

[29] Vazzoler A. E. A. M. (1996). *Biologia da reprodução de peixes teleósteos: teoria e prática*.

[30] Kawakami E., Vazzoler G. (1980). Método gráfico e estimativa de índice alimentar aplicado no estudo de alimentação de peixes. *Boletim do Instituto Oceanográfico*.

[31] Hahn N. S., Fugi R. (2007). Alimentação de peixes em reservatórios brasileiros: alterações e conseqüências nos estágios iniciais do represamento. *Oecologia Brasiliensis *.

[32] Hyslop E. J. (1980). Stomach contents analysis—a review of methods and their application. *Journal of Fish Biology*.

[33] Hellawell J. M., Abel R. (1971). A rapid volumetric method for the analysis of the food of fishes. *Journal of Fish Biology*.

[34] Fonteles-Filho A. A. (1989). *Recursos Pesqueiros: Biologia E Dinâmica Populacional*.

[35] Hurlbert S. H. (1978). The measurement of niche overlap and some relatives. *Ecology*.

[36] Novakowski G. C., Hahn N. S., Fugi R. (2008). Diet seasonality and food overlap of the fish assemblage in a pantanal pond. *Neotropical Ichthyology*.

[37] Gotelli N. J., Entsminger G. L. (2006). *Ecosim: Null Models Software for Ecology*.

[38] Entsminger G. L. (2012). *Journal of Waste Conversion, Bioproducts and Biotechnology*.

[39] Albrecht M., Gotelli N. J. (2001). Spatial and temporal niche partitioning in grassland ants. *Oecologia*.

[40] Townsend C. R., Begon M., Harper J. L. (2006). *Fundamentos em Ecologia*.

[41] Nikolsky G. V. (1963). *The Ecology of Fishes*.

[42] Nikolsky G. V. (1969). *Theory of Fish Population Dynamics*.

[43] Wootton R. J., Potts G. W., Wootton R. J. (1984). Introduction: tactics and strategies in fish reproduction. *Fish Reproduction: Strategies and Tactics*.

[44] Andersson M. (1994). *Sexual Selection*.

[45] Hunt J., Breuker C. J., Sadowski J. A., Moore A. J. (2009). Male-male competition, female mate choice and their interaction: determining total sexual selection. *Journal of Evolutionary Biology*.

[46] Bagarinao T., Thayaparan K. (1986). The length-weight relationship, food habits and condition factor of wild juvenile milkfish in Sri Lanka. *Aquaculture*.

[47] Gurgel H. d. (2004). Estrutura populacional e época de reprodução de *Astyanax fasciatus* (Cuvier) (Characidae, Tetragonopterinae) do Rio Ceará Mirim, Poço Branco, Rio Grande do Norte, Brasil. *Zoologia*.

[48] Stephens P. R., Wiens J. J. (2009). Bridging the gap between community ecology and historical biogeography: niche conservatism and community structure in emydid turtles. *Molecular Ecology*.

[49] Punzalan D., Hosken D. J. (2010). Sexual dimorphism: Why the sexes are (and are not) different. *Current Biology*.

[50] Gause G. F. (1932). Experimental studies on the struggle for existence. I. Mixed populations of two species of yeast. *Journal of Experimental Biology*.

[51] Hardin G. (1960). The competitive exclusion principle. *Science*.

[52] Nokman J. R. (1928). The South American characid fishes of the subfamily with Serrasalmoninae a revision of the genus. *Proceedings of the Zoological Society of London*.

[53] Jégu M., Dos Santos G. M. (1988). Une nouvelle espèce du genre *Mylesinus* (Pisces, Serrasalmidae), *M. paucisquamatus*, décrite du bassin du rio Tocantins (Amazonie, Brésil). *Cybium*.

[54] Romanuk T. N., Hayward A., Hutchings J. A. (2011). Trophic level scales positively with body size in fishes. *Global Ecology and Biogeography*.

[55] Gerking S. D. (1994). *Feeding Ecology of Fish*.

[56] Hahn N. S., Loureiro V. E., Delariva R. L. (1999). Atividade alimentar da curvina *Plagioscion squamosissimus* (Heckel, 1840) (Perciformes, Sciaenidae) no rio Paraná. *Acta Scientiarum Biological Sciences*.

[57] Zavala-Camin L. A. (1996). *Introdução aos Estudos Sobre Alimentação Natural em Peixes*.

[58] Hölldobler B., Wilson E. O. (1990). *The Ants*.

[59] Kronfeld-Schor N., Dayan T. (2003). Partitioning of time as an ecological resource. *Annual Review of Ecology, Evolution and Systematics*.

[60] Sazima I., Machado F. A. (1990). Underwater observations of piranhas in western Brazil. *Environmental Biology of Fishes*.

[61] Agostinho C. S., Hahn N. S., Marques E. E. (2003). Patterns of food resource use by two congeneric species of piranhas (*Serrasalmus*) on the upper paraná river floodplain. *Brazilian Journal of Biology*.

[62] Winemiller K. O. (1989). Ontogenetic diet shifts and resource partitioning among piscivorous fishes in the Venezuelan ilanos. *Environmental Biology of Fishes*.

[63] Pompeu P. S., Godinho H. P. (2003). Dieta e estrutura trófica das comunidades de peixes de três lagoas marginais do médio São Francisco. *Águas, peixes e pescadores do São Francisco das Minas Gerais*.

[64] Raposo R. D. M. G., Gurgel H. D. C. B. (2003). Variação da alimentação natural de *Serrasalmus spilopleura* Kner, 1860 (Pisces, Serrasalmidae) em função do ciclo lunar e das estações do ano na lagoa de Extremoz, Rio Grande do Norte, Brasil. *Acta Scientiarum Biological Sciences*.

[65] Marçal-Simabuku M. A., Peret A. C. (2002). Alimentação de peixes (osteichthyes, characiformes) em duas lagoas de uma planície de evundação brasileira da bacia do rio paraná. *Interciência*.

[66] Hynes H. B. N. (1970). *The Ecology of Running Waters*.

[67] Matthews W. J. (1998). *Patterns in Freshwater Fish Ecology*.

[68] Fernandes I. M., Henriques-Silva R., Penha J., Zuanon J., Peres-Neto P. R. (2014). Spatiotemporal dynamics in a seasonal metacommunity structure is predictable: the case of floodplain-fish communities. *Ecography*.

